# Spontaneous and inherited *TP53* genetic alterations

**DOI:** 10.1038/s41388-021-01991-3

**Published:** 2021-08-13

**Authors:** Arnold J. Levine

**Affiliations:** Institute for Advanced Study, Princeton, NJ USA

**Keywords:** Cancer genetics, Cancer genetics

## Abstract

The p53 protein is a transcription factor that prevents tumors from developing. In spontaneous and inherited cancers there are many different missense mutations in the DNA binding domain of the *TP53* gene that contributes to tumor formation. These mutations produce a wide distribution in the transcriptional capabilities of the mutant p53 proteins with over four logs differences in the efficiencies of forming cancers in many diverse tissue types. These inherited and spontaneous *TP53* mutations produce proteins that interact with both genetic and epigenetic cellular modifiers of p53 function and their inherited polymorphisms to produce a large number of diverse phenotypes in individual patients. This manuscript reviews these variables and discusses how the combinations of *TP53* genetic alterations interact with genetic polymorphisms, epigenetic alterations, and environmental factors to begin predicting and modifying patient outcomes and provide a better understanding for new therapeutic opportunities.

## Introduction: the origins and development of spontaneous cancers

### Cellular targets of the genetic alterations of cancers: tissue-specific stem cells (TSSCs)

A large collection of evidence indicates that every organ in our body contains TSSCs, which are capable of replicating themselves or committing to differentiate into many or all of the cell types that make up the organ. Among the first clear demonstrations of these TSSCs were hematopoietic stem cells (CD−34+ cells), which can produce many of the blood cells and some tissue-associated cells in several organs [1]. These stem cells begin their existence in the embryonic yolk sac, move to the fetal liver, and then reside in the bone marrow throughout the rest of life. The colon stem cell is termed the slim cell. It resides in the crypt of the colon [2] and replicates to produce a large number of cell types that make up the colon. The epidermal stem cells are responsible for producing skin and reside near the hair follicles [3]. These and other TSSCs have a number of properties in common. Each type of TSSC replicates symmetrically (reproducing more stem cells) increasing or replenishing the pool of TSSCs. These stem cells are duplicated in response to signals from the Wnt pathway, employing frizzled receptors that regulate the levels of beta-catenin, which in turn regulate both cell adhesion molecules (Cadherins) and act in the nucleus as a heterodimer with TCF-4 (T-cell factor 4) to transcribe a series of Wnt-regulated genes. The expression of this pathway is further enhanced by extracellular factors termed the R-spondins, which act upon G-protein coupled receptors, increasing Wnt induced transcriptional activity. The G-linked receptors, LGR-4, 5, 6 (leucine-rich repeat) are found in ectodermal and endodermal derived TSSCs (skin and colon) as well as some mesenchymal stem cells (blood cells) [4]. After a pool of TSSCs are formed in an organ, some of them are selected to differentiate, altering their epigenetic marks by producing progenitor cells or intermediates in the developmental pathway forming the tissue. The body of a normal human will produce billions of new cells each day. Some of this comes from tissue repair, but the majority contributes to the varying turnover of tissues; the half-life of colon cells is about four days [5]; the half-life of skin tissue is about twenty-eight days [6], and blood cells turn over continuously [1].

This large amount of replication of many different TSSCs in their niches or compartments of the body over a lifetime results in the accumulation of mutations in the TSSCs, which then compete with each other for reproductive fitness, or propagation, of clones of stem cells. Weissman [1] has called these stem cells “units of natural selection for tissue formation, for germ line development and cancer development”. The mutation rate in these tissue-specific stem cells has been estimated at about 20–60 mutations per year of life using whole-genome sequencing of cloned organoids produced from single stem cells in culture. The stem cells were obtained from individuals aged 3–87 years old and from several different organs [7] and single-cell DNA sequencing was also performed [8]. When these mutations impact genes of TSSCs in pathways for cell cycle reproduction, cell death, or DNA damage repair the stem cells can be selected for reproductive fitness and they can compete to take over the pool of TSSCs in an organ. These experiments predict that over a lifetime TSSCs will accumulate clones with mutations that are precursors to a series of mutations that lead to the development of cancers. Deep DNA sequencing from normal tissues of individuals has identified such clones of cells with mutations in oncogenes or tumor suppressor genes [9, 10]. Mutations that give rise to a clonal expansion of a TSSC in a tissue have been termed the initial, or truncal mutation, in a pathway leading to the development of clonal expansion and the start of development of cancer.

### Selectivity of expression or function of oncogene or tumor suppressor gene mutations in TSSCs

There are hundreds of oncogene mutations that can contribute to cancer development and tens to hundreds of tumor suppressor gene mutations that can permit cancer development. Based upon the frequency of each oncogene or tumor suppressor gene mutation in different spontaneous cancerous tissues, and therefore different TSSCs, there is a clear preference for one or another gene mutation in different tissues (see the TCGA as an example [11]). This is even more striking with inherited tumor suppressor gene mutations such as *RB1, TP53, PTEN, BRCA1 or BRCA2*, etc. [9, 10]*. BRCA-1 and 2* are expressed and employed for homologous DNA repair in many cell and tissue types of the body, yet mutations in these genes give rise to ovarian and breast cancers at a higher incidence than many other tissues. Elledge and his colleagues [12] have pointed this out and explored the reasons why the same mutated oncoproteins expressed in different tissue lineages not only can give rise to cancers in one tissue type but not the other but also the same mutant protein (*B-RAF*^*V600G*^) present in colon cancers and melanomas respond to inhibitors in the latter but not in former cancer. They have pointed out that the different epigenetic states in different tissues and TSSCs have an impact upon mutational selection, and the signal transduction pathways that contain these mutated genes are structured differently, with or without feedback loops or additional components.

### The order of mutations in oncogenes and tumor suppressor genes in TSSC determines the properties and the age of onset of cancers

Based upon the conclusions derived from studies such as those discussed above, the initial, or truncal mutation, spontaneously occurring or inherited in a TSSC exerts a positive selection for reproductive fitness in a cell population of TSSCs [9, 10]. This results in a clonal expansion of that stem cell, and so a second mutation is more likely to occur in that clone of TSSCs containing the first mutation. If the second mutation has no further impact upon the fitness of the cell, then it will not contribute to the development of cancer, but if it adds to the fitness of the TSSC, then it expands the cell number and target size for additional mutations. Over the past several years a large literature has accumulated demonstrating the presence of such mutations in selected genes in normal or benign tissue that has clonally expanded in a local area of tissue over a lifetime [13], in skin and esophagus for example [14, 15], where a calculated reproductive fitness with *NOTCH1* mutations and *Tp53* mutations can be quantified [16]. Similarly, the expansion of myeloid precursors in the blood over time, and treatment with mutagens gives rise to CHIP (clonal hematopoiesis of indeterminate potential), a benign expansion of myeloid cell numbers with selected mutations at reproducible frequencies (*DNMT3A, TET-2, ASXL-1, JAK-2, SF3B1,* and *TP53*) [17–19].

Clearly, mutations occur randomly over time. However, the selection of a mutation, so as to confer an added reproductive fitness upon the cell and its progeny, inserts an order to the mutational progression from benign to malignant cancers. This was first shown in three different and independent ways employing colorectal cancers. Vogelstein and his colleagues obtained benign and malignant tumors of the colon from colonoscopies employing a number of human patients. The smallest polyps that were obtained contained a mutation in the adenomatous polyposis coli gene (*APC*). Slightly larger but still benign polyps had the *APC* mutation and a *KRAS* mutation. Even larger benign polyps obtained from patients had the *APC* mutations, a *KRAS* mutation, and a *SMAD* mutation (in the TGF-beta pathway). Finally, malignant colorectal cancers had all three of the previous mutations plus mutations in both alleles of the *TP53* gene [20, 21]. Vogelstein inferred an order in these mutations from the sizes of the tumors and the benign to malignant transition. In a second study, Sato and Clevers started with normal human colorectal tissue-specific stem cells in organoid cultures and introduced selected mutations in the *APC, KRAS, SMAD-4*, and *TP53* genes in different orders and combinations, employing CRISPR-Cas-9 [22]. These experiments demonstrated that the most efficient way to produce a malignant tumor by adding mutations was in the same order observed by Vogelstein. In the third set of experiments, this time carried out in mice, Jenkins and Copeland initiated colon cancers by turning on a transposon that either activated oncogenes or inactivated tumor suppressor genes by random insertion into the genome. They observed that it took a long time (about 100 days) to obtain these tumors in all the mice under study. When the Tp53 gene was inherited in the germline and then the transposon was activated it took 80 days; when *Smad4* was inherited it took 60 days; when *KRAS* was inherited it took 50 days, and when *APC* was inherited it took 25 days to produce colorectal cancers [23]. Rather clearly, all three experiments agree that colorectal cancers are formed, most commonly, by an ordered selection of random mutations, and individuals will develop colorectal cancer earlier in their lifetime if they inherit a truncal, or initial mutation, in this case APC, first in a specific gene order in a TSSC. The efficiency of tumor formation is determined by an ordered selection for reproductive fitness, resulting in a series of benign clonal expansions, until the last mutation creates a malignant tumor. The random nature of mutations gives rise to a tremendous diversity of mutations in a tumor so as to obscure the ordered progression of the mutated genes essential for cancer formation.

## Inherited *TP53* mutations and The Li-Fraumeni syndrome

**Fig. 1:**
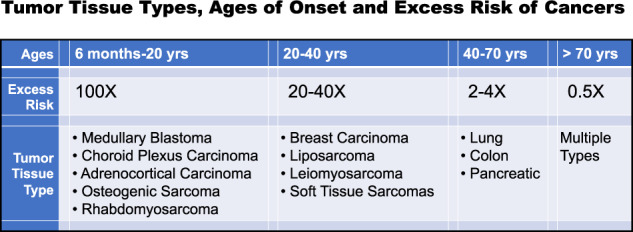
Tumor tissue types, ages of onset, and excess risk of cancers. Li Fraumeni patients inherit a mutant TP53 allele that acts at an early age of onset (six months to 20 years) indicating that the mutant p53 protein functions as an initial or truncal mutation promoting benign cell replication and clonal expansion and producing a high excess risk for cancer. TP53 mutations that give rise to cancers later in life more likely function as a later mutation in an ordered series of mutant genes that drive cancerous growth. By 50–70 years of life, cancers arise with low excess risk and the mutant p53 protein acts in the benign to malignant transformation as the last gene in the ordered series of cancer forming mutations [9, 10, 27].

How can we determine what mutations in tumor suppressor genes are the initial or truncal mutations giving rise to an expanded clonal number of mutant TSSCs for each tissue-specific cancer? By definition, germline mutations in tumor suppressor genes are the truncal or initial mutations in a TSSC that will give rise to cancer at a young age [9, 10]. Because the inherited mutation occurs in every TSSC type in the body, the preferential tissue-specific phenotypes of the cancers formed to provide evidence for selection and clonal expansion of that mutation in a TSSC shortly after birth. Three phenotypes of the tumors arising in these patients determine the nature of the TSSC that has a functional initial or truncal Tp53 mutation that initiates tumor formation: (1) A very early age of onset of the tumor formation, (2) the tumor tissue type that is produced at this very early age indicates the TSSC and its clonal expansion, and (3) the excess risk of the tumor tissue type in Li-Fraumeni Syndrome patients compared to the general population. Figure 1 presents these phenotypes for tumor tissue types most commonly produced over the lifetime of a Li-Fraumeni patient with a germline Tp53 mutation. Rather clearly, the ages at which specific tumor tissue types are produced fall into three categories: six months to 20 years, 20–45 years, 45–70 years. At the youngest ages are medullary blastomas, choroid plexus carcinomas and papillomas, adrenocortical tumors, rhabdomyo sarcomas, and osteogenic sarcomas. In the 20–45-year old category are breast tumors in females, brain tumors (gliomas and glioblastomas), and soft tissue sarcomas, and the 45–70-year old group develops leiomyosarcomas, colon, lung, and pancreatic cancers. In some individuals with germline TP53 mutations over the age of 70, no cancers are detected over their lifetimes, and the incidence of cancers in Li-Fraumeni patients with *TP53* mutations over 70 years old falls to a rate below the general population, which is increasing dramatically after 60–70 years of age [24–27]. The excess risk for Li-Fraumeni patients to develop a tumor also varies with age and tissue type (the TSSC). From 6 months to 20 years of age there is about a 100 fold excess risk to develop those tissue-specific tumors. From 20–40 years of age there is about a 20–40-fold excess risk, and from 50–70 years of age the excess risk ranges from about 2–4-fold. Above 70 years of age, the excess risk falls to below 0.5 times [27]. The single most common cancer of patients with Li-Fraumeni Syndrome is female breast cancer. This mostly occurs in women between the ages of 20–40 years, which is earlier than carriers of *BRCA-1* and *-2* mutations [28]. It is notable that the tumor types observed at the youngest ages with the highest risks derive from the ectoderm or neuro-ectoderm. From 20–40 years of age, with a high to intermediate excess risk, tumors are predominately from mesodermal tissue, and in the lower risk older age group from 50–70 they derive from endoderm. The TSSCs that derive from these first three germ layers of stem cells suggest that initial or truncal mutations acting first in a series of mutations occur in TSSC that form epithelial and neuro-ectodermal tissue types. By contrast, Tp53 mutations of endodermal derived TSSC are functionally active last in the series of mutations, as observed in colon, ovary, pancreatic, and lung cancers, and confer a malignant phenotype upon a previously benign tumor with several mutations. This is identical to a set of observations made in the mouse experiments of Jenkins and Copeland discussed above [23]. It predicts that the spontaneous cancers with TSSCs derived from endoderm (prostate, high grade serous ovarian cancers, colorectal, non-small cell lung cancers) produce malignant tumors by acquiring a Tp53 mutation late in the process of cancer formation [9, 10].

## The domain structure and functions of the p53 protein

**Fig. 2:**
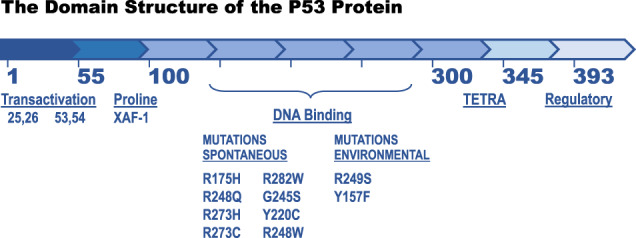
A linear representation of the p53 protein with five domains. The XAF-1 protein binds within the proline-rich domain (see Fig. 2) and the great majority of missense mutations that contribute to cancers reside in the DNA binding domain. The eight spontaneous mutations and two environmental mutations form the ten most common TP53 mutations (33% of cancers), which have very little or no transcriptional activity. More than 350 additional TP53 missense mutations have a weak transcriptional activity, a much lower frequency of occurrence in producing cancers over a four log distribution, and cause 67% of cancers. The hypothesis that pro-apoptotic XAF-1 binding to a weak transcriptional p53 protein promotes apoptosis so that the weak allele fails to form cancer. An XAF-1 gene transcriptionally silenced by epigenetic marks or a polymorphism that inactivates the protein would then permit weak, minor TP53 alleles to produce cancer [37].

The human p53 protein is a transcription factor that regulates a series of genes forming a large interconnected pathway [10]. The human p53 protein is composed of 393 amino acids commonly numbered from the N-terminus-1, to the C-terminus-393. The protein is composed of five structural and functional domains (Fig. 2). Amino acids 1–55 include two transactivation regions (amino acids 21–28 and amino acids 47–55) [29, 30] each of which regulates some similar and some different sets of genes. These amino acid sequences attract and assemble RNA polymerases and chromatin-modifying enzymes that open the chromatin for transcription and add protein modifications to the polymerase and p53 protein, helping to direct which genes are transcribed. Amino acids 55–100 form a proline-rich domain with repeated sequences of PXXP (P representing proline and X any amino acid). This is a protein-protein interaction domain that regulates cell growth and apoptotic efficiency [31, 32]. Amino acids 100–300 form a sequence-specific DNA binding domain that imparts upon the p53 protein the specificity to identify genes it regulates. There are hundreds of different missense mutations in this domain and they differ from each other in their DNA binding efficiencies, loss of transcriptional gene functions, and even possible gain of function mutations [33–35]. The frequencies with which each of these hundreds of different mutations occur in all cancer types differs by up to four orders of magnitude. The loss of DNA binding, transcriptional efficiency, and frequency of each of these mutations are strongly correlated [33–35]. The fourth domain at amino acids 320–345 is the tetramerization domain of the p53 protein. The p53 transcription factor forms from two dimers producing a tetramer that binds to 20 base pairs or two turns of the DNA helix [36]. Some mutations in this domain can also cause familial and spontaneous cancers, but these tend to have lower penetrance and a weaker cancer-causing phenotype [37]. The fifth domain can regulate transcriptional activity. It contains seven lysines whose epsilon amino group can be acetylated to enhance transcription or methylated to inhibit transcription in some stem cells [38, 39]. Deletion of this domain, or phosphorylation of it, enhances DNA binding. These properties are reviewed in Fig. 2.

**Fig. 3:**
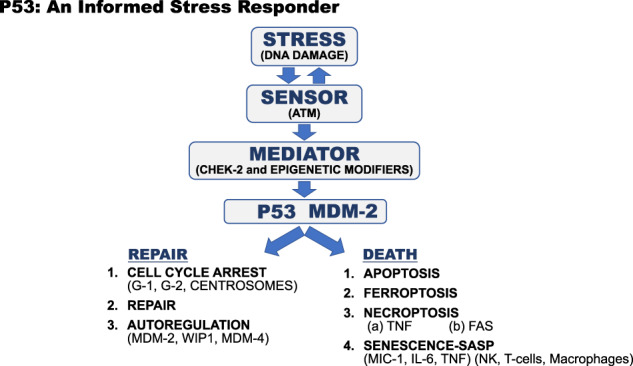
P53: an informed stress responder. The p53 and MDM-2 proteins form a module in the cell where p53 transcribes the MDM-2 gene and the MDM-2 protein promotes the degradation of the p53 protein. Stress signals, in this case, DNA damage is sensed by the ATM protein which phosphorylates a CHEK-2 mediator which in turn phosphorylate serine -15 in the p53 protein, and other sites in p53 and MDM-2, that disrupts the MDM-2-P53 protein complex. This increases p53 protein levels and along with other epigenetic modifications of p53 and MDM-2 the p53 transcription factor makes an informed choice between cell cycle arrest, DNA repair, and return to homeostasis or programed cell death by one of five possible methods [10].

The function of the p53 protein is to respond to a wide variety of intrinsic and extrinsic cellular stresses. Stress is defined here as any interference with an orderly progression of cellular functions or division. In response to many different types of stresses (Fig. 3 highlights a DNA damage stress), epigenetic modifications or signals are sent from a stress detector (ATM) through a stress mediator (CHEK2) to the p53 (serine -15) protein and MDM2 protein, which is the E3 ubiquitin ligase regulating the instability of the p53 protein by poly-ubiquitination. The inhibition of the MDM2 activity (along with other proteins that function with MDM2) increases the half-life of the p53 protein within minutes of the stress occurring. The half-life of the p53 protein increases from minutes to hours and the epigenetic modifications plus the increased concentrations of the p53 protein activate transcription of a selected set of genes (Fig. 3). The epigenetic modifications provide information about the nature and intensity of the stress, which determines the response (transcriptional program) that may be either cell cycle arrest and repair of the damage or cell death.

The properties of the p53 pathway include:

1. The gaining of epigenetic information about the nature and intensity of stress.

2. The making of a decision about which transcriptional program will be employed to respond to the stress.

3. The p53 pathway is very redundant in how it mediates cell cycle arrest at G-1 and at G-2.

4. The pathway can make any of five choices of the types of cell death it employs to kill cells.

5. The pathway is extensively connected to many other pathways (metabolic, immunological, the cell cycle, DNA damage repair, ribosome biogenesis, cell death and differentiation, etc.) [10].

These five properties of the p53 pathway give rise to the observation that mutations in the *TP53* gene are the most common genetic alteration in human cancers. Understanding the loss and/or the gains of functions of the p53 mutant protein is essential to an understanding of the origins and properties of diverse cancers.

## The frequencies of spontaneous and inherited Tp53 mutations in humans with and without cancers

As previously discussed, there are hundreds of different *TP53* missense, nonsense, deletion, frameshift, and insertion mutations in the *TP53* genes sequenced from individuals with spontaneous and inherited cancers [33–35]. Some of these mutations tend to be tissue-specific because they are brought about by different known mutagens in the environment (34,35, see Fig. 2). About 20–25% of them are true loss of function mutations (nonsense, deletions, frameshifts), but the missense mutations, which represent 75–80% of the mutations, occur at different frequencies (over four orders of magnitude) in all tissue types of human cancers [33–35]. These different missense mutations often have different phenotypes that are based upon diverse DNA binding efficiencies, transcription of different genes, temperature-sensitive properties, and possible gain of functional phenotypes. The ten most frequent *TP53* mutations in cancers are present in 33% of the cancers (Fig. 2). The remaining hundreds of other *TP53* missense mutations are found (more than ten independent times) in 67% of cancers. The top ten most frequent *TP53* mutant alleles (Fig. 2) have common features. They are very poor at transcribing p53 specific genes [33–35] and some mutations are at the DNA binding contacts of the protein while others change the structure and melting temperatures of the p53 protein [35]. Attempts are now underway to classify different mutant alleles that are inherited and to explore the penetrance of different *TP53* mutations, their ability to show any tissue specificity (expression or function or loss of function in TSSCs), or other phenotypes.

The great majority of individuals who inherit *TP53* genetic alterations are identified by virtue of developing specific tumor types at a very young age and then having their *TP53* gene sequenced, or because they are related to patients from identified Li-Fraumeni families. These individuals and families are estimated to occur in the general population at about 1/20,000 people, which is not too different from the frequency of mutations in other tumor suppressor genes occurring with fairly high penetrance.

K. de Andrade, P. Hainaut, M. Achatz, and S. Savage [40, 41] and others have explored sequencing databases composed of unrelated individuals not selected for cancer history. Then they ask what the frequencies of *TP53* germline mutations in the remaining group are, so as to eliminate the bias of having been diagnosed with specific cancers at a very young age. Surprisingly, in these databases the prevalence of carriers of potentially pathogenic germline *TP53* mutations varies from 1/500–1/5,000 persons compared to the previous estimated incidence of 1/20,000 who are first identified because of cancer. Although these prevalence estimates are dynamically altered based on variable mutation classifications and highly impacted by some specific controvertible spontaneous *TP53* mutations, the databases employed could introduce other possible biases, such as CHIP, or enrichment of mutations potentially associated with lower cancer penetrance or with phenotypes not typically associated with LFS [17, 18]. With some uncertainty, these initial numbers bring up the possibility that there may be environmental or genetic suppressors of *TP53* mutations that lower cancer penetrance or weaken the phenotype. A weak *TP53* mutant allele, defined as being at very low frequencies in individuals with either spontaneous or inherited cancers, could be at that low frequency because of genetic and/or environmental factors. As a larger number of these individuals with *TP53* mutations, but no cancerous phenotypes are identified, the frequency, nature, and properties of each allele will be recorded, and possible genetic suppressors or environmental history could help to explain the phenotype. The identification of such genetic or environmental suppressors is valuable, in that the identified genes, or environmental activity, can be useful in developing therapeutic approaches to treatments.

## Genetic modifiers that could be of interest

There is an excellent and detailed review of *TP53* polymorphisms acting as genetic modifiers of p53 related phenotypes that demonstrate the complexities of multi-genic or even multi-allelic variations upon cancer phenotypes [42]. What follows is a brief overview of these polymorphisms and some additional observations that reinforce the importance of this topic, both at the basic and clinical levels of understanding Tp53-related cancers.

### *XAF-1* (XIAP associated factor-1) gene and protein

**Fig. 4:**
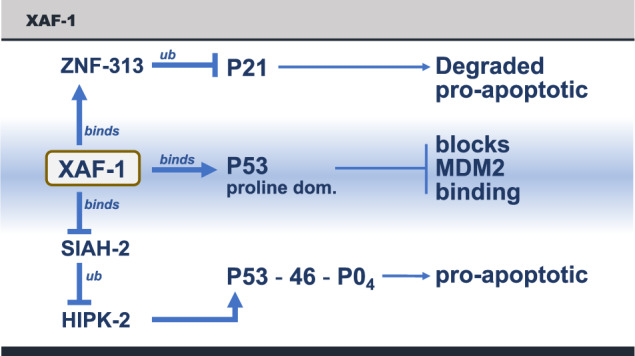
The functions of the XAF-1 protein in promoting p53 mediated apoptosis. 1. The XAF-1 protein binds to the p53 protein in the proline domain and dissociates MDM-2 from p53, stabilizing p53. 2. The XAF-1 protein binds to the ubiquitin ligase SIAH-2, which inhibits its functions, increasing the levels and activity of HIPK-2, which in turn phosphorylates p53-serine 46, which promotes the transcription of a pro-apoptotic p53 pathway. 3. The XAF-1 protein also binds to ZNF-313, a ubiquitin ligase that promotes the degradation of the p21 protein (cell cycle arrest). This is also is a pro-apoptotic activity [46].

The XAF-1 protein is a 33.1 Kda protein whose gene is located on human Chromosome 17, just two mega-bases away from the *TP53* gene. The protein has seven zinc fingers, and was first shown to bind to the X-linked inhibitor of apoptosis (XIAP) and inactivate it, promoting apoptosis [43]. The *XAF1* gene is positively regulated for transcription by IRF-1 (interferon regulatory factor-1) promoting TNF mediated cell death; XAF1 transcription is negatively regulated by HSF-1 (heat shock factor-1) [44]. The gene has been classified as a tumor suppressor that is commonly shut down at the level of transcription by heavily methylated chromatin [45]. In addition to these regulatory functions, the XAF1 protein acts at three different p53 intersecting pathways to increase p53 levels in the cell, resulting in the transcription of several p53 regulated genes whose proteins promote apoptosis (Fig. 4). First, the XAF1 protein strongly promotes p53-mediated apoptosis by the binding of XAF1 to the proline domain of p53, which in turn competes off the binding of the MDM-2 protein, which is the E3-ubiquitin ligase for p53. This stabilizes the p53 protein, increasing its half-life and concentration [46]. In addition, the proline-rich domain is known to impart growth-inhibiting and pro-apoptotic activity to the p53 protein [29, 30]. Second, the XAF1 protein binds to and inhibits the ubiquitin ligase SIAH-2, which in turn, stabilizes the HIPK2 (homeobox interacting protein kinase -2) that now phosphorylates serine 46 of the p53 protein, promoting the transcription of several p53-regulated pro-apoptotic genes (*APAF-1, BAX, NOXA, PUMA, FAS-1, TNFα*) [46]. Third, the XAF1 protein binds to the E3 ubiquitin ligase ZNF-313, which poly-ubiquitinates the p21 protein that becomes degraded, and this favors an apoptotic response (see Fig. 4). This is a remarkable diversity of activities that focus the p53 protein to drive apoptosis and tumor suppression [46].

Drs. Pinto and Zambetti have extended these results by demonstrating the role of the *XAF*-*1* gene product in the formation of cancers [37]. It turns out that the *XAF-1* gene contains a polymorphism (rs146752602), E134*, which places a chain termination codon in the 134th amino acid of the *XAF*-*1* gene, inactivating the protein. This occurs in about 1/125 individuals in the population. In southern Brazil, where this study [37] was carried out, there is a large population of carriers of the *TP53* mutation R337H (this population originally arose from a genetic founder effect), which is localized in the tetramerization domain. The cohort understudy had 203 unrelated patients who developed cancers, 582 family members, and 42,000 newborns (the p53 mutation is carried in the heterozygous state). In that cohort, there was a clear enrichment for the *XAF*-*1* E134* and *TP53* R337H compound genotype among the individuals who developed sarcomas (*p* = 0.003). There was an even greater enrichment of the *XAF*-*1*, E134* genotype in individuals with second and third malignancies (*p* = 0.006). Consistent with these clinical findings, the levels of m-RNAs from p53 transcriptionally regulated genes were significantly lower in primary fibroblasts expressing *TP53*-R337H mutant protein with the *XAF*-*1* 134* allele compared to those with the *XAF*-*1* wild type allele. The levels of transcription mediated by the *TP53* R337H allele is lower than *TP53* wild-type alleles, but higher than the top 10 *TP53* mutant alleles (Fig. 2), which are largely transcriptionally inactive. This demonstrates an epistatic genetic relationship between the *XAF*-*1* gene and the diverse types of wild-type and mutant *TP53* alleles [37].

Pinto and Zambetti proposed a very interesting hypothesis [37]: individuals who, with functional, but attenuated *TP53* mutant alleles, like R337H, will have a higher risk for tumors when they also have an E134* mutation in the *XAF*-*1* gene compared to the *XAF*-*1* wild type allele because p53 does not mediate apoptosis efficiently without XAF-1. Inactivating *TP53* mutations that eliminate *TP53* transcriptional activity (e.g., the hot spot mutations) would not be affected by the *XAF*-*1* E134* mutation (epistasis). However, weakly transcribing spontaneous *TP53* mutations will be stimulated by the wild type XAF1 protein, killing the nascent tumor, but the same weak *TP53* allele will produce a tumor when paired with an *XAF*-*1* E134* polymorphism, which could enhance the frequencies of this class of *TP53* mutations in the spontaneous and inherited cancers. This suggests the possibility that the very different frequencies of weak mutant *TP53* alleles observed in cancers arise from a wild-type *XAF*-*1* gene being present and able to enhance p53 mediated cell death. Thus, some cancers, like those (with) R337H p53 mutations, will enrich for the presence of the *XAF*-*1* E134* allele or other polymorphic alleles that might inactivate the enhancer of apoptosis [37].

### *TP53*Pro 47 Ser, (rs1800371) polymorphism

The proline-47 to serine amino acid polymorphism in the *TP53* gene arose in an African haplotype, is found in 6% of Africans, and is present in 1% of African Americans [47]. Given the discussion above, where phosphorylation of serine-46 is required for an efficient p53 apoptotic pathway, it is understandable that serine-47 in the p53 protein impairs apoptosis after most, but not all, DNA damage [47]. The Serine-47 polymorphism results in reduced levels of phosphorylation of serine-46 after many types of DNA damage, chemotherapy, and radiation. This variant of p53 is defective in the ability to regulate genes involved in the sensitivity of cells to ferroptosis, a process of iron- and lipid peroxide-mediated cell death. This ferroptotic defect is caused by increased anti-oxidant accumulation in serine-47 cells [48]. This leads to iron accumulation in mouse and human cells containing the serine-47 variant, and indeed this variant is associated with a disease called “iron overload” in individuals of African descent [49]. Serine-47 also is associated with increased cancer risk in mice and humans and with decreased sensitivity of tumor cells to many chemotherapeutic drugs [47]. However, the cisplatin class of drugs and BET inhibitors (Bromo-domain and extra terminal motif) do induce apoptosis in tumors with serine-47 [50, 51], demonstrating remarkable specificity between the use of therapies and the genotype of the Tp53 gene and its protein. Interestingly, the increased glutathione accumulation in serine-47 mice causes increased activity of mTOR, a major regulator of metabolism [52]. Consequently, serine-47 mice are larger and more physically fit than control littermates, suggesting that this variant may have been selected for at one time [52].

### *MDM2* SNP309 (rs2279744) polymorphism (a T to G change in the promoter/enhancer region of the MDM-2 gene)

The MDM-2 protein is the major E-3 ubiquitin ligase for the regulation and degradation of the p53 protein [53]. The p53 protein helps to transcribe the MDM-2 gene, such that these two proteins form an auto-regulatory loop, keeping each other in a stable concentration range unless stress or a drug inhibits the loop [54]. The levels of the p53 protein are regulated by protein degradation (a rapid response), whereas the levels of the MDM-2 protein are predominantly regulated by transcription (a slow response). 5′ to the start of the MDM-2 gene is an enhancer element that binds transcription factors like p53. In that region of the DNA, there is a common polymorphism termed SNP 309, where either a T or a G residue resides. The Sp-1 transcription factor binds much better to the G-residue than to the T residue within this element and so makes more *MDM2* mRNA transcripts and more MDM-2 protein. Women carriers of germline *TP53* mutations and diagnosed with Li-Fraumeni Syndrome and a SNP-309 G residue develop breast cancers with a mean age of 29 years, whereas women with a SNP-309 T-residue develop breast cancers with a mean age of 39 years (*p* = 0.01) [55]. These observations are not controlled for the *TP53 mutant* allele, nor for other polymorphisms, which could differ among patients. However, when SNP309 enhancer regions with the G or T alleles regulating the *Mdm2* gene were engineered into the germline of mice, the G-allele produced elevated levels of Mdm2 leading to reduced p53 protein and decreased apoptosis [56]. Consistent with these findings, the SNP309 G allele accelerated tumor onset and tumor spectrum in mice harboring a *p53* hot spot mutation compared to those with the SNP309 T allele [56].

### *TP53* Arg 72 Pro polymorphism

Humans evolved in Africa and the Tp53 gene is almost entirely composed of a proline residue at codon 72 in the present African population. The polymorphic change from proline to arginine occurred sometime during the migration out of Africa, so that Caucasians in Europe and the United States are up to 40% homozygous arginine. This polymorphism has been shown to have an impact upon the decision to program cell cycle arrest or cell death, and responses to nutrient deprivation [57]. There is some indication that the p53 arginine form has an increased affinity for MDM-2, resulting in lower levels of p53 and earlier ages of onset of tumors in carriers of germline TP53 mutations and Li-Fraumeni families [28].

### PIN3 a 16base pair duplication in intron 3 of the *TP53* gene

It is possible that this polymorphism is protective, with first cancers in Li-Fraumeni families occurring at older ages [26].

### PAS, (rs78378222) a *TP53* polyadenylation signal 3′ UTR polymorphism

The normal polyadenylation signal for *TP53* mRNA is AATAAA, whereas the PAS polymorphism is AATA**C**A, which leads to less *TP53* mRNA and protein in a cell. The PAS SNP is associated with increased levels of prostate cancers (OR = 1.44), gliomas (OR = 2.35), and colorectal adenocarcinomas (OR = 1.39), and the SNP is strongly associated with cutaneous basal cell carcinoma [58].

Several of the SNPs (polymorphisms) discussed above are in strong linkage disequilibrium when measured in selected populations (African decent and Caucasians) and environments: SNPs in introns 2 and 3 along with codon 47 (proline to serine) and codon 72 (proline to arginine) demonstrate positive linkage disequilibrium. This suggests that these SNPs have co-evolved and define complex haplotypes that are found in populations that evolved in different geographic localizations. It is likely, therefore, that these sets of SNPs (haplotypes) are composed of genetic variants that interact or cooperate to express specific functional properties that either optimize stress responses and/or tumor suppression in response to environmental variations, as first suggested by P. Hainaut [27].

## Some questions remain to be explored

### What are the functions of the *TP53* gene and protein that are important to prevent cancers?

The p53 gene and protein are only present in multicellular animals, not in plants, bacteria or yeast. The origins of this gene have been traced back to a common ancestor of choanoflagellates and humans, some 600–800 million years ago [59]. In all invertebrates the *TP53* protein DNA binding domain binds to the same DNA sequence it binds to in humans. Furthermore, the vertebrate and invertebrate orthologues of the p53 protein regulate similar genes for cell death in response to genomic damage [59]. This conservation of protein sequences, DNA binding sequences and regulated gene functions suggest a real importance for multicellular animal life processes. The second reason why *TP53* is important to study is that genetic alterations in the *TP53* gene are the most common mutations observed in cancers of humans [10, 11, 33, 34].

These two observations suggest that the answer to the question, “what p53 regulated gene protects us against cancers” would be useful to ask experimentally. Attempts to test this question have not led to the uncovering of a single important gene for tumor suppression. Rather, it seems more likely that the answer is in the structure and functions of the entire p53 pathway and its properties discussed in this article. The redundancies in the pathway allow it to function in spite of destructive mutations eliminating multiple pathways for enforcing cell death. In addition, the method or process of cell death chosen by the p53 protein sensing a stress (Fgure 3) could impact the nature and types of immune response to antigen presentation by dying cells. If redundancy is such an important feature, why do simple single missense mutations in the *TP53* gene itself, disrupt the entire pathway? Why didn’t evolution select for redundancies in *TP53* like functions producing many *TP53* like genes to back it up? Some answers to this and related questions are explored elsewhere [10].

### What is the mutational profile, or signature, of genetic alterations in cells with *TP53* mutations? Is this a clue to why it is central to cancer production?

The p53 protein regulates the number of centrosomes that duplicate at each cell division and permit accurate segregation of chromosomes [60]. The loss of this p53 function results in a failure to synthesize p21 and thus cyclin E is no longer under p53 control. Both p21 and cyclin E bind to centrosomes, and the subsequent overexpression of cyclin E gives contributes to multiple centrosomes and subsequent aneuploidy. 1. There are losses and gains in chromosomes and dramatic changes in gene copy number. 2. The loss of p53 functions results in deletions and gene amplification, both by loss of entire chromosomes and by local DNA loss and gene amplifications, 3. chromothripsis, the fragmentation of chromosomes and the reassembly of the parts in a novel order, and the formation of circular DNA and double minute chromosomes.

These types of mutations result in high levels of cell death, selection for cells that replicate with abnormal gene copy numbers, divide and are selected for fitness, metastasis, gene amplification, and hyper- and hypodiploid genotypes. Chromosomal and gene copy number abnormalities are commonly lethal in normal cells, perhaps because the cells are killed by *TP53* functions that sense the abnormality, but the loss of *TP53* seems to permit the toleration of copy number abnormalities. The mechanisms behind the tolerance of abnormal gene copy numbers are not well understood. These phenotypes form the signature of *TP53* loss of function. Perhaps the restoration of p53 functions in a cancer cell will restore the lethality of abnormal gene copy numbers and eliminate cancer.

### Are there genetic, epigenetic, and environmental modifiers of *TP53* mutations?

An abundance of genetic and epigenetic modifiers of p53 function, are highlighted in this review. There are several interesting conclusions. Epigenetic modifiers can change the age of onset of tumors, the elimination of tumors by weak *TP53* mutant alleles by apoptosis, and even the frequency of tumor types and tissue types. There is a strong developmental impact upon *TP53* gene expression or function in different TSSCs derived from ectoderm, mesoderm, and endoderm. Different environmental carcinogens (cigarette smoke, lung cancers and codon 157 mutations; aflatoxin, liver cancers and codon 249 mutations) have been shown to act upon the *TP53* gene mutating specific bases and codons in specific tissues that result in tissue-specific tumor types (Fig. 2). There are clearly genetic, epigenetic and environmental modifiers of the Tp53 gene and p53 protein functions.

### What is the relationship between epigenetic alterations and the p53 protein functions and responses?

The first line of evidence that large epigenetic changes were one of the intrinsic stresses that are sensed by and activate p53 was demonstrated by Jaenisch and his colleagues [61]. They created a conditional knockout of the DNA methyl-transferase gene-1 (*Dnmt1*) in a CRE-Lox mouse. The Dnmt1 enzyme is a cytosine methyltransferase that adds a methyl group to cytosines in new strands of DNA at GpC locations opposite methyl-CpG residues in the template strand. Jaenisch knocked out this gene in cells derived from that mouse and those cells divided two times, producing an unmethylated double-stranded DNA, and then these cells died by apoptosis [59]. A knock-out of the p53 genes in these cells resulted in no cell death and the cells went on to be transformed and were tumor producing. Thus, the p53 protein sensed the unmethylated DNA and killed the cell by apoptosis. This result was confirmed by Yamanaka and his colleagues [62]. They added four transcription factors to fibroblasts in cell culture (Oct-4, Sox-2, Klf-4, and c-Myc), and at a very low frequency, (a few percent) in weeks or months, the cells dramatically reduced and reprogramed their methylated CpG residues, changing the epigenetic state and producing induced pluripotent stem cells [62]. When this was done with cells containing a temperature-sensitive mutant *p53*, at the non-permissive temperature (p53 inactive), the transcription factors c-Myc and Klf-4 could be eliminated and the percentage of IPSC produced went up as high as 80% and the kinetics of producing IPSC occurred in days, not weeks or months, all in a temperature-sensitive fashion, proving it was p53 that regulated these processes [63, 64]. These experiments demonstrate that the p53 protein can sense a dramatic change in the levels of genomic methylated CpG residues, and in response, kill these cells by apoptosis. One of the stresses that activate p53 is a change in genomic epigenetic marks. Azacytidine and decitabine are incorporated in place of cytidine in DNA but do not permit methylation of the cytosine residues. Curiously, these two drugs are much more efficient in killing cells with *Tp53* mutations than they are in killing cells with wild-type *TP53* [65]. That is also true for cells with mutant and wild-type *TP53* that are tumorigenic in animals [66] and humans [67].

How p53 senses changes in the epigenome, and perhaps in the chromatin that packages the genome, or the transcription factors that interact with the genome remains unclear. The *TP53* gene and its protein appear to be both the guardian of the genome (Fig. 3) and of the epigenome, ensuring fidelity with a penalty of death. That is a regulatory mechanism that a multicellular organism can live with. It seems likely that changes in CpG methylation in the genome change with age, are predictive of a healthy longevity and differ between males and females [68]. There are numerous suggestions in the literature that histone methyltransferases and demethylases play a role in the sexual dimorphism that impacts life span, cancer incidences, autoimmunity, immune responses to infectious diseases, metabolic differences and even cognitive differences [69–71]. We are just starting to learn about the mechanisms that regulate this sexual dimorphism [72]. It is clear that p53 interfaces with every one of these pathways and phenotypes, perhaps through epigenetic marks, and a better understanding of these processes is our next challenge.
